# Compact Spatial Heterodyne Spectrographs for Future Space-Based Observations: Instrument Modeling and Applications

**DOI:** 10.3390/s24144709

**Published:** 2024-07-20

**Authors:** Ayan Sahoo, Joice Mathew, Andrew Battisti, Brad Tucker

**Affiliations:** 1 Department of Physical Sciences, Indian Institute of Science Education and Research Kolkata, Mohanpur 741246, India; 2 Advanced Instrumentation and Technology Centre, Research School of Astronomy and Astrophysics, Australian National University, Canberra, ACT 2611, Australia; 3 Research School of Astronomy and Astrophysics, Australian National University, Canberra, ACT 2611, Australia; andrew.battisti@anu.edu.au (A.B.); brad.tucker@anu.edu.au (B.T.); 4 ARC Centre of Excellence for All Sky Astrophysics in 3 Dimensions (ASTRO 3D), Australia

**Keywords:** high resolution spectroscopy, compact spectrograph, SHS, space payload, instrument modelling

## Abstract

High-resolution spectroscopy employing spatial heterodyne spectrographs (SHS) holds significant promise for forthcoming space missions, building upon its established track record in science applications. Notably, it offers exceptional performance and cost- effectiveness in the ultraviolet-visual (UV-Vis) region compared to contemporary instruments. SHS instruments provide high-resolution capabilities and substantially larger etendues than similar resolving power instruments. This study introduces a comprehensive Python-based SHS model integrated with a user-friendly web scraping interface for target star selection, parameter generation, and 2D interferogram creation. Our SHS model demonstrates double the resolving power of a grating spectrometer and a throughput comparable to a Fourier transform spectrometer (FTS) but without moving parts, enhancing robustness for deployment in space. The interferogram processing algorithm includes flat-fielding, bias removal, apodization, and an inverse Fourier transform (IFT) for accurate spectrum retrieval. Despite bandwidth limitations due to resolving power constraints, SHS models excel in applications requiring high spectral resolution over narrow wavelength ranges, such as studying isotopic emission lines. The model provides optimization results and trade-offs for system parameters, ensuring precise spectral recovery with realistic signal-to-noise ratio (SNR) values. SHS is versatile and effective for various scientific applications, including investigating atomic and molecular emissions from comets, planetary atmospheres, the Earth’s atmosphere, the Sun, and the interstellar medium (ISM). This research significantly contributes to expediting the development and deployment of SHS instruments, demonstrating their potential across numerous scientific domains.

## 1. Introduction

High-resolution spectroscopy of faint extended objects in the UV-Vis spectrum is challenging due to the trade-off between sensitivity and resolving power. While low-resolution spectroscopy reveals basic parameters like composition and intensity, high-resolution spectroscopy provides detailed insights into velocity, temperature, pressure, and isotopic signatures [1,2,3]. These are pivotal in various astronomical domains, including the search for extraterrestrial life. Fourier transform spectrometers (FTS) are preferred for high-resolution spectra from faint extended sources because of their large etendue and precision optics [4,5]. However, FTS instruments perform better in the infrared range because the maximum sampling interval in terms of OPD (optical path difference), as stated by Nyquist sampling theorem, must be $\Lambda_{min}/2$ ($\Lambda_{min}$ being the minimum wavelength in the available bandpass for the instrument), which is very challenging to achieve at shorter wavelengths (UV-Vis) [6]. They are also typically bulky, making them less suitable for spaceborne applications. In contrast, spatial heterodyne spectroscopy (SHS) instruments [5], which also use Fourier transforms, are effective across all wavelengths, including UV-Vis, owing to the heterodyne mixing they perform. SHS systems are lightweight, compact, and offer flexible alignment [7]. These benefits make SHS ideal for studying faint extended sources such as Hα emissions from star-forming regions, atmospheric emission lines, and planetary auroras [8], and UV-Vis sources in general [9,10,11,12,13].

**Figure 1:**
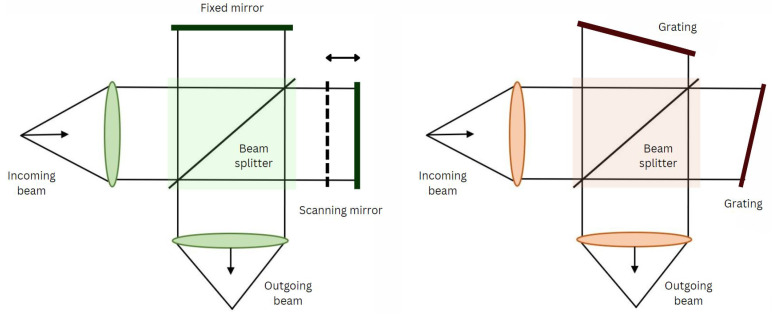
On the left-hand side is the schematic for the MI, which incorporates two mirrors and a beam splitter. One of the mirrors is moved to record spatial (or, equivalently, temporal) data. On the right-hand side is the schematic for SHS, which is almost the same as MI, but it uses gratings rather than mirrors, and the gratings are tilted at the Littrow angle.

At its core, the SHS model employs a modified Michelson interferometer (MI) configuration [14], replacing traditional moving mirrors with diffraction gratings (Figure 1).

**Figure 2:**
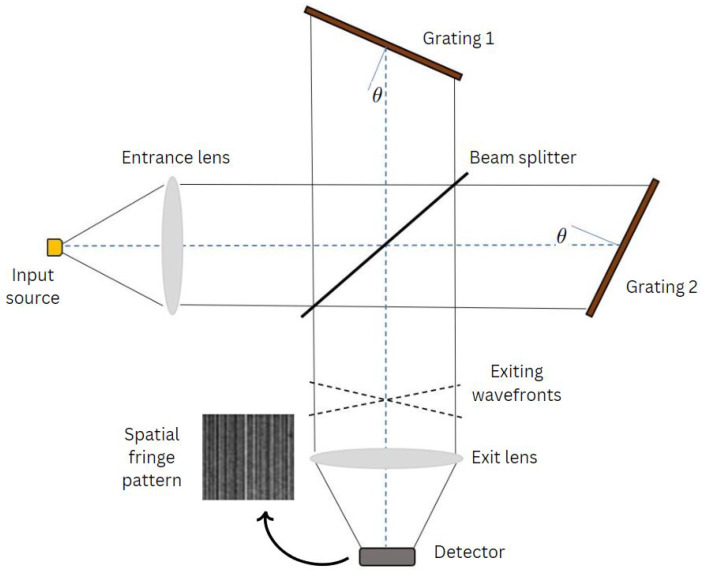
The SHS schematic diagram: For each wavelength in the incident wavefront, two wavefronts with a wavelength-dependent crossing angle between them exit the interferometer. The resulting superposition of Fizeau fringes with wavelength- dependent spatial frequencies are localized near the gratings and imaged by exit optics on a position-sensitive detector. The image is the Fourier transform of the input spectrum about the heterodyne wavelength (the wavelength producing parallel output wavefronts) [17].

These gratings introduce a spatially dependent phase shift on incident light, forming an interferogram that encodes spectral information. A slight tilt in one of the gratings induces spatial modulation within the interferogram, facilitating spectral data retrieval through Fourier transformation ((Figure 2) shows this structure in detail). SHS’s compact and robust design makes it well-suited for space-borne applications where durability and efficiency are paramount [15]. Moreover, due to the spatial fringes formed by the tilt of the gratings, SHS offers rapid data acquisition by simultaneously recording the entire interferogram and eliminating the need for sequential scanning characteristics of conventional spectrometers [16]. Additionally, its adaptability to different spectral ranges enhances its versatility, catering to a wide array of scientific inquiries. However, SHS does face challenges, including the need for sophisticated algorithms and substantial computational resources to interpret intricate interference patterns accurately. SHS instruments also face a multiplex disadvantage where noise from each spectral element sampled in the bandpass is distributed across the entire interferogram. High-quality imaging detectors are essential for data processing. Additionally, spatial heterodyning can introduce cross-talk between spectral channels, requiring careful calibration.

This paper introduces an SHS model for simulating 2D interferograms, demonstrating spectral recovery and parameter generation to gain insight into the physical scales of the instrument and enable analysis across a wide range of spectral types. The tools provided aid analysis and design optimization for future missions, demonstrating spectral recovery with expected signal-to-noise ratio (SNR) values.

We present the simulation results for a typical use case to demonstrate the model’s performance. We provide input parameters like bandpass and required resolving power along with the spectra to generate the output parameters, the 2D interferogram, and retrieve the calibrated spectra along with the expected SNR. We also show the results from a parameter optimization study to help users fine-tune the parameters according to the required science case.

## 2. Fundamentals of a Spatial Heterodyne Spectrograph

**Figure 3:**
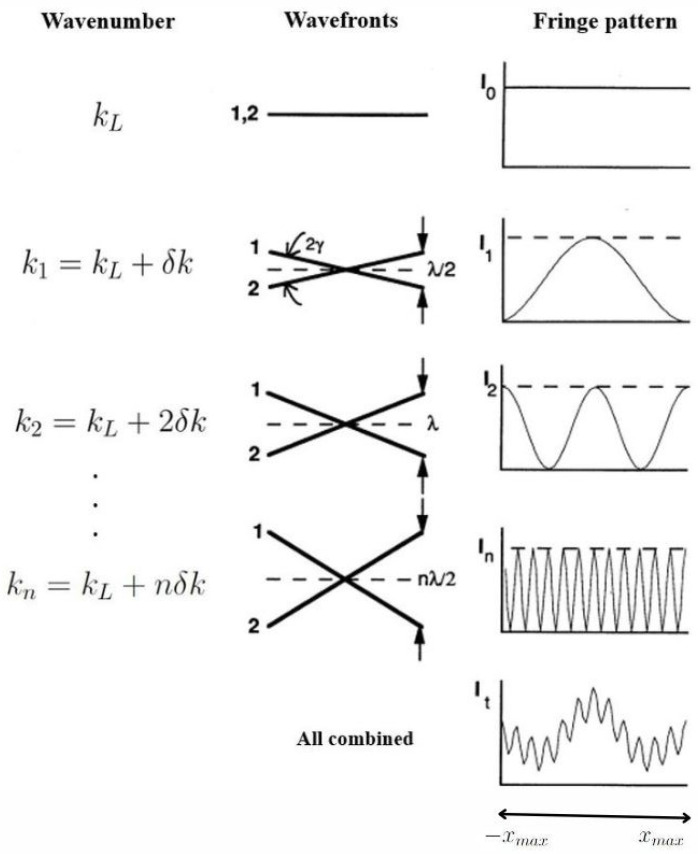
Relationship between wavenumber, wavefronts, and intensity patterns on the detector produced by SHS systems. The wavefronts are crossed at angles $2\gamma ≃4\left(1-\frac{k_{L}}{k}\right)\tan\theta_{L}$ where $k_{L}$ and $\theta_{L}$ are the Littrow wavenumber and grating angle, respectively. In the second row is a spectral line with intensity $I_{1}$ and a wavenumber $k_{1}$, that is δk from $k_{L}$ produces crossed wavefronts with a maximum separation of $\frac{\lambda }{2}\left(=\frac{1}{2k_{L}}\right)$ at the edges of a detector [18].

The SHS separates the different wavenumber components (or called spectral components) of the input light via the diffraction grating equation, as shown in (Figure 3):

(1) $$k\left[\sin\left(\theta_{L}\right)+\sin\left(\theta_{L}-\gamma \right)\right]=m/d$$

where *k* is the wavenumber of the incident light, $\theta_{L}$ is the Littrow angle (a property of the diffraction gratings), γ is the angle that the output wavefront makes with the normal to the detector, *m* is the order of diffraction, and 1/d is the density of the grating grooves. If γ equals zero, the observed wavenumber is the Littrow wavenumber [5]. We can solve for the Littrow angle of the gratings if we know the Littrow wavenumber:

(2) $$\theta_{L}=\sin^{-1}\frac{m}{2dk_{L}}$$

For the SHS setup, the equation for interference takes the form

(3) $$I\left(x\right)=\int_{0}^{\infty }I\left(k\right)[1+Cos\left(2\pi \left(\nu_{F}\cdot x\right)\right]\delta k$$

where

$$\nu_{F}=4\left(k-k_{L}\right)\tan\theta_{L}$$

where $k_{L}$ is called the Littrow wave number and is a characteristic of our grating.

However, there is a limitation imposed by the above equation. This equation, which is for a one-dimensional interferogram, cannot differentiate between two frequencies that are equally greater and smaller than the Littrow wavenumber $k_{L}$. The SHS samples the absolute value of the wavenumber offset that is $\left|k-k_{L}\right|$. This could create a problem because then we would be able to sample the wavevectors with a wavenumber on only one side of the Littrow wavenumber. We will discuss a workaround in Section 2.2.

### 2.1. Etendue, Resolving Power, and Field of View Advantages of the SHS

The principal advantages of using an SHS are due to its high resolving power capabilities while maintaining high etendue. This high etendue results from its very high field of view (FOV) compared to other high-end instruments with similar resolving powers (Table 1).

The maximum field of view (Ω) of SHS is defined by its resolving power (*R*), as shown below [18];

(4) $$\Omega =\frac{2\pi }{R}$$

One thing to note here is that the formula above treats FOV as the case when the SHS is kept in an open, mechanically collimated mode [19].

When using it in association with a telescope, we have to make sure that the FOVs of the two match, which will result in decreases from the maximum collimated. But there is no net change in etendue as the decrease in FOV is balanced out by the increase in the collimating area of the telescope. The main takeaway is that when observing a target with an extent larger than the maximum FOV, there is no use for a telescope. But if the extent is smaller than the maximum, then a telescope can be a significant improvement. One problem is that the usable bandpass of an SHS instrument is inversely related to the resolving power. Thus, we have to keep the resolving power to a level such that the bandpass is broad enough to be used for the science case of interest.

### 2.2. Two-Dimensional Interferogram of SHS

The workaround to the problem regarding a one-dimensional interferogram and spectral recovery discussed previously is to introduce a small tilt in the SHS mirrors (Figure 2) by a pitch angle $\frac{\phi }{2}$ perpendicular to the plane of interference. This is a standard yet very powerful technique that has been used in many SHS applications over the years [13]. The tilt results in a small symmetric rotation of the wavefronts in each arm of the interferometer, producing a low-resolving power vertical fringe pattern $f_{y}=\phi k$ with a frequency that changes only slowly in the vicinity of the heterodyne wavenumber and is unresolved over a small bandpass. This results in fringes from wavelengths on opposite sides of Littrow being rotated in opposite directions; wavenumbers $k>k_{L}$ are rotated clockwise, while $k<k_{L}$ are rotated counterclockwise. The two-dimensional interference intensity pattern corresponding to a multicomponent spectral source is then given by [3]

(5) $$I\left(x\right)=\int_{0}^{\infty }I\left(k\right)[1+Cos\left(2\pi \left(\nu_{F}\cdot x+f_{y}\cdot y\right)\right]\delta k$$

We can also determine the necessary diffraction grating width for a desired number of interferogram samples, *N*, using the Nyquist theorem, which says that to effectively detect a wave signal using a finite number of detectors, one needs to sample data at least twice that of the highest-frequency fringes in the sample [20]:

(6) $$W=\frac{N}{2\left(4\Delta k\right)\sin\theta_{L}.},$$

where $k_{max}-k_{L}=k_{L}-k_{min}=\Delta k$ denotes the highest detectable spatial fringe frequency.

Using this result, we determine the maximum position *x* on the detector:

(7) $$x_{\max}=\frac{1}{2}W\cos\theta_{L}$$

The spectral resolution:

(8) $$dk=\frac{1}{2\left(4\left(\tan\theta_{L}\right)x_{\max}\right)}=\frac{2(\Delta k)}{N}.$$

And the interferogram sample spacing:

(9) $$dx=\frac{2x_{max}}{N}=\frac{1}{4\left(\tan\theta_{L}\right)Ndk}$$

Thus, the resolving power can be expressed as

(10) R=k/dk=4Wksinθ≃2·m·N

which is twice that of conventional grating spectrometers.

Further, we can see that in comparison to a 1D interferogram, a 2D interferogram lets us use twice the spectral bandwidth for an exact resolution (or resolving power) without any ambiguity in wavenumber. That is a significant advantage, given that the resolution of the SHS instrument is constrained by its usable spectral bandwidth. We used these equations to generate the output parameters from the input parameters in Section 5.

## 3. Simulation of the SHS Model and Generating a 2D Interferogram

**Figure 4:**
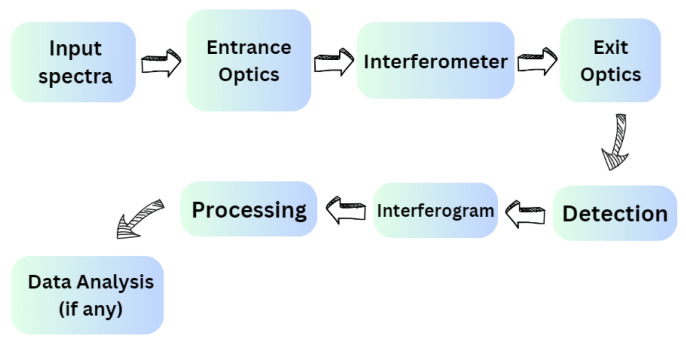
Fundamental structure of the model: we provide input spectra and generate parameters. Then, the spectral data go through the entrance optics part, followed by the interferometer and exit optics segment. Finally, the generated 2D interferogram is detected, and processing is performed to generate SNR plots and recover the inputted spectra.

We provide tools to assist with design optimization characterization and data analysis. Possible instrument design parameters and assumptions are used to model the SHS and generate raw interferograms in **Python**. An interferogram processing algorithm converts the raw interferograms to calibrated radiance spectra. The model (Figure 4) serves as a tool for SHS design parameters and a system and performance analysis tool for any SHS design based on the user’s specific requirements. The model takes in the following parameters as input:

1. minimum accepted wavenumber, $k_{\min}$ or maximum accepted wavelength $\Lambda_{\max}$.
2. maximum accepted (Littrow) wavenumber, $k_{\max}$ or minimum accepted wavelength $\Lambda_{\min}$.
3. required resolution, R.
4. diffraction grating order, m.
5. diffraction grating groove density, d.

### 3.1. Entrance Optics

The entrance optics block in a real instrument includes an optical aperture and a collimating lens, defining the instrument’s FOV. Material properties introduce self-emission and optical transmission terms, where self-emission, represented as a blackbody radiator with unit emissivity and temperature T (can be a free parameter, but as a case we have considered 290K) is added to the scene radiance. The FOV, a constant, can be factored out subsequently.

The self-emission is calculated using Planck’s equation, as follows:

(11) $$L_{self}\left(k\right)=\frac{2h\nu^{3}}{c^{2}}\frac{1}{\exp\left[\frac{h\nu }{k_{B}T}-1\right]},$$

where the wavenumber, *k*, has units of ${\mathrm{cm}}^{-1},k_{B}$ is Boltzmann’s constant, and *T* is the temperature of the blackbody. Finally, the transmission $\tau_{1}\left(k\right)$ of the entrance optics is assumed to be wavenumber-dependent.

We model the transmission with data for calcium fluoride lenses available on the Thorlabs website [21].

The output of the entrance optics [22] is, therefore,

(12) $$L^{\prime }\left(k_{n}\right)=\Omega \cdot \tau_{1}\left(k\right)L_{\mathrm{scene}\;}\left(k_{n}\right)+L_{\mathrm{self}\;}\left(k_{n},T_{1}\right)$$

where Ω is the system FOV.

### 3.2. Interferometer Block

In the interferometer block, the optical elements are the beam splitter and the two gratings.

We must account for the diffraction grating efficiencies and the transmission function associated with the beam splitter. The effective radiance is now

(13) $$L^{\prime \prime }\left(k\right)=\tau_{BS}^{2}\left(k\right)\left(\frac{1}{2}\eta_{A}\left(k\right)+\frac{1}{2}\eta_{B}\left(k\right)\right)L^{\prime }\left(k\right)$$

where $\eta_{A}$ and $\eta_{B}$ are the efficiencies of diffraction gratings A and B, respectively, and $\tau_{BS}$ is the wavenumber-dependent transmission function of the beam splitter.

Therefore, the interferogram calculated in the interferometer block using Equation (5) is

(14) $$I\left(x\right)=\sum_{n}\frac{1}{2}L^{\prime \prime }\left(k_{n}\right)[1+Cos\left(2\pi \left(\nu_{F}\cdot x+f_{y}\cdot y\right)\right]\delta k,$$

$\nu_{F}$ and $f_{y}$ hold the same meaning as discussed in the previous section. *x* and *y* are the discrete variables for a position on the detector, both ranging from $-x_{max}$ to $+x_{max}$ and are changed by δx as we model a square array of pixels on the detector.

For beam splitters, we also use the data available for commercially available beam splitters on the ThorLabs website [21] and obtain the required data using interpolation.

### 3.3. Exit Optics

The exit optics take up the high-resolution interferogram thus produced. It includes a second transmission function and another self-emission term, modeled as a blackbody (here, temperature T can be a free parameter, but just for instance, we have used 280K). The output of the exit optics is then

(15) $$I^{\prime }\left(x\right)=\tau_{2}{\left(k\right)}_{avg}I\left(x\right)+\sum_{n}\tau_{2}\left(k\right)L_{\mathrm{self}}\left(k_{n},T_{2}\right)dk,$$

where $\tau_{2}{\left(k\right)}_{avg}$ is the mean of the exit optics transmission.

The first term on the left-hand side corresponds to the effect of the optical elements on the interferogram, and the second term is due to the black body self-radiation from those optical elements.

To obtain the data for $\tau_{2}$, we do the same as $\tau_{1}$ (see Section 3.1).

### 3.4. Detection

Even though the system generates a high-resolution interferogram, which can have arbitrarily small δx, the detector takes up data according to the number of user-defined samples, which correspond to the number of pixels available on the sensor to detect the interferogram produced. Thus, we divide the whole interferometer area according to this and then find the interferogram samples at those positions using interpolation of high-resolution sample data.

$$I^{\prime }\left(x\right)\to I_{resampled}^{\prime }\left(x\right)$$

In addition, in a realistic detector, we need to take care of the variation in the sensitivity of the pixels across the sensor, which is expressed as $S_{FPA}$, where FPA stands for focal plane array.

(16) $$I^{\prime \prime }\left(x\right)=S_{FPA}\left(x\right)I_{resampled}^{\prime }\left(x\right)$$

We model $S_{FPA}$ using a random but uniform distribution between the values 0.999 and 0.985 (as most scientific grade CCDs have a variation between 1–2% [23]). One may also try to model it using a Gaussian distribution of pixel sensitivities.

### 3.5. Incorporating the Noise into Our Model

We treat $I^{\prime \prime }(x)$ as a statistical distribution for the incoming photons in the system. Therefore, we normalize $I^{\prime \prime }(x)$ to obtain the distribution and count the total number of photons as

(17) $$I_{p}=\frac{I^{\prime \prime }\left(x\right)}{\sum I^{\prime \prime }\left(x\right)}$$

$$n_{total}=\int L^{\prime \prime }\left(k\right)dk.$$

We proceed to multiply $n_{p}$ with the statistical distribution

$$n\left(x\right)=I_{p}\cdot n_{total}$$

We term n(x) as photon count along the x-axis on the screen Now, we add the noises to the photon count as follows:

$$n^{\prime }\left(x\right)=n\left(x\right)+n_{noise}$$

$n_{noise}$ consists of the contributions of shot, read, and dark noise.

Finally, we normalize it again to get the modified statistical distribution perturbed by noises,

(18) $$n_{p}=\frac{n(x)}{\sum n(x)}$$

And finally, the resultant noisy interferogram that is reported is estimated to be

(19) $$I^{\prime \prime \prime }\left(x\right)=n_{p}\cdot \sum I^{\prime \prime }\left(x\right)$$

## 4. Interferogram Processing Algorithm

### 4.1. Flat Fielding

We use the flat fielding process to correct the interferometric data for variations in inter-pixel sensitivity and dark noise. These variations are usually constant in the detectors and are taken care of only after the instrument is built.

The method that we employ here uses two measurements of the same source—one made with one interferometer arm blocked, and the second made with the opposite arm blocked. We divide our interferograms collected using the unit-normalized mean of the two single-arm measurements. The The flat-field corrected interferogram [24] is then

(20) $$I_{ff}\left(x\right)=\frac{I(x)}{{\left(\frac{I_{A}\left(x\right)+I_{B}\left(x\right)}{2}\right)}_{norm}}$$

where *I*(*x*) is any measured interferogram and $I_{A}\left(x\right)$ and $I_{B}\left(x\right)$ are the measurements of the known source taken while blocking arms B and A, respectively.

The denominator rescales the interferogram according to each pixel’s sensitivity and dark noise.

### 4.2. Bias Removal

The measured interferogram is the sum of a modulated and unmodulated term. The modulated time is the interferogram, by definition, whereas the unmodulated time may be neglected. We determine the interferogram bias (or the unmodulated term) by averaging all the samples in the interferogram. Averaging removes all the periodic wave-like components (in our case, the EM waves of different frequencies), as they sum up to a value of zero over one full period. Finally, we subtract this from the measured interferogram to leave us with only the modulated term.

### 4.3. Apodization

Mathematically, the interferogram *I*(*x*) is an infinite array along the x-axis, and all of the terms must be considered to recover the spectra perfectly. But in reality, the interferogram produced in an instrument (in this case, our simulation) is finite, and thus errors related to truncation creep in. Truncating the interferogram leads to non-zero amplitude data points at its ends. This truncation causes spectral energy transfer from lower to higher frequencies during the inverse Fourier transform (IFT) [20].

Reducing the impact of leakage caused by abrupt detector cutoff or signal truncation involves smoothing the truncation at the ends. This process lessens the multitude and amplitudes of the side lobes [22]. To achieve this, the finite, discontinuous interferogram is multiplied by an apodization function, namely, the Hamming window [25], gradually decaying to zero at the ends.

### 4.4. Inverse Fourier Transform

Performing the inverse Fourier transform gives us 2D spectra. We need to extract the part that contains the actual data. Thus, we take the y-axis array with the maximum intensity and proceed.

The IFT gives us complex numbers as output, of which we should take only the magnitude [20], as we want the IFT of the interferogram to be real and symmetric.

(21) $$L_{recov}\left(k\right)=\sqrt{L_{recov,R}^{2}\left(k\right)+L_{recov,I}^{2}\left(k\right)}$$

It is necessary to take the magnitude of the IFT rather than throw the imaginary part away, because we want to maintain the spectrum power.

### 4.5. Noise Removal

To reduce noise in the spectrum after IFT of the interferogram, it is common to use filters like mean and median [20]. The mean filter smooths the signal by averaging nearby samples. In contrast, the median filter, better at preserving edges, is more suitable for spectral signal processing as it retains the emission and absorption lines. Thus, our model employs a median filter for noise reduction.

### 4.6. Calibration

While modeling our SHS instrument, we considered many external factors that altered our recovered spectra concerning the original model spectra ($L_{og}$) we used. Thus, we had to remove these effects by using a simple mathematical expression [22] as follows,

(22) $$L_{recov}\left(k\right)=a\left(k\right)\left[L_{og}\left(k\right)+b\left(k\right)\right]$$

where *a*(*k*) is termed system responsivity and b(k) is the system offset. We use two black bodies for calibration. If the respective radiance measurements of the hot and cold BBs are $L_{H}\left(k\right)$ and $L_{C}\left(k\right)$ at temperatures, $T_{H}$ and $T_{C}$, the responsivity is

(23) $$a\left(k\right)=\frac{L_{H}\left(k\right)-L_{C}\left(k\right)}{L_{BB}\left(T_{H},k\right)-L_{BB}\left(T_{C},k\right)}$$

where $L_{BB}\left(TH,k\right)$ and $L_{BB}\left(TC,k\right)$ are the expected BB radiation measurements of a hot and cold BB at temperatures TH and TC, respectively. The instrument offset is then

(24) $$b\left(k\right)=\frac{L_{C}\left(k\right)}{a(k)}-L_{BB}\left(T_{C},k\right)$$

Finally, the measured and calibrated spectral radiance is,

(25) $$L_{cal}\left(k\right)=\frac{L_{recov}\left(k\right)}{a(k)}-b\left(k\right)$$

## 5. SHS Modeling Application for Stellar Astronomy

The model we have developed is intended to foster studies in SHS and its applications. It is expected to help in the parameter selection and design optimization of future space missions involving SHS. In addition, we have also employed a web-scraping interface to select a star of the required spectral type from the Pickles Atlas [26] and obtained the system parameters required for observing the source represented by the input spectrum using an SHS instrument.

Any input parameter should work in the model. As a realistic scenario, keeping the physical limitations of a low-cost Cubesat mission in mind, we used a set of input parameters, which have been listed in (Table 2). Using the mathematical equations shown in Section 2 and the input parameters, the model generated output parameters, which have been listed in (Table 3). These output parameters were then used to run the program in **Python**, generate and demonstrate the interferogram and SNR plots, and retrieve the spectra to analyze the performance. We carried out a test run of our simulation of the SHS model. We used data from the Pickles Atlas [26], namely the file named **pickles_10.fits**, which corresponds to a spectral type A2V (A-type main-sequence star). The following subsections show the model and interferogram processing algorithm results using this SHS design (the Pickles Atlas provide spectra in flux density units, i.e., power per unit area per unit wavelength. Thus, while using the SHS model on it, we divided by the known FOV of the Pickles instrument, which is 20 arc sec by 10 arc sec [27] or 200 arc sec squared and then multiplied by the system FOV of 1000 arc sec square which the user can further change. If the user uses spectra in specific intensity (power per unit area per unit wavelength per unit steradian) units, one can directly multiply with the system FOV.) (corresponds to a main sequence star). The user inputs for the test run were as follows:

### 5.1. Spectral Recovery

**Figure 5:**
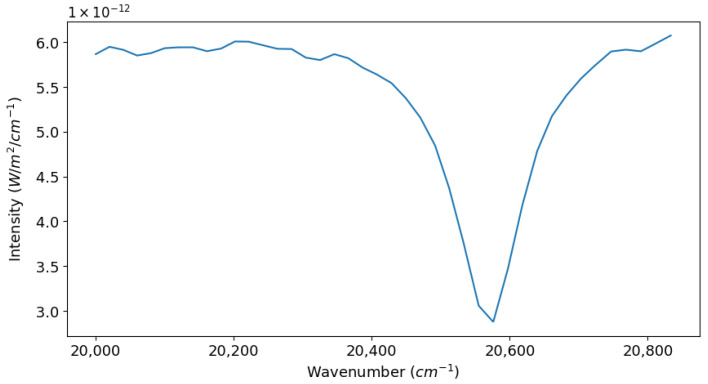
Input spectrum we used as a science case for demonstrating the SHS model. The spectral feature shown here corresponds to the Hβ line typical to A-type main sequence stars.

**Figure 6:**
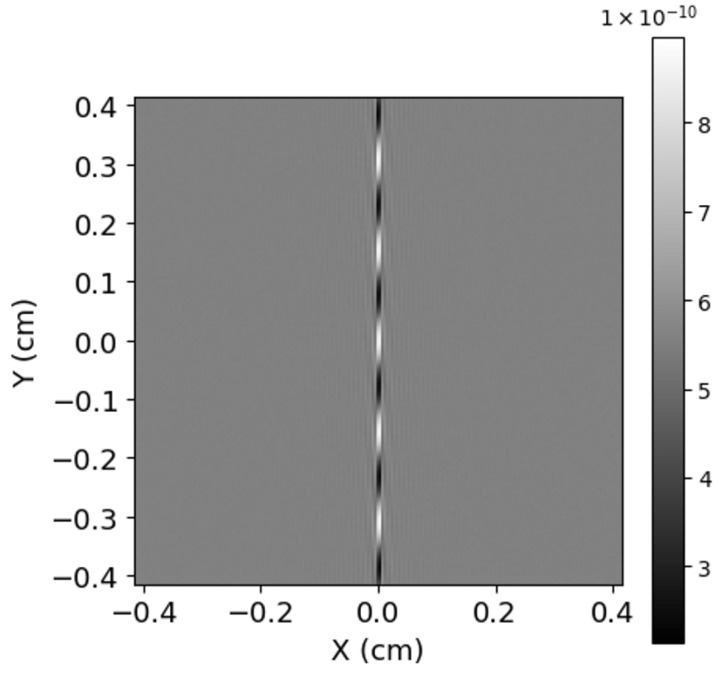
2D Interferogram generated by the model.

**Figure 7:**
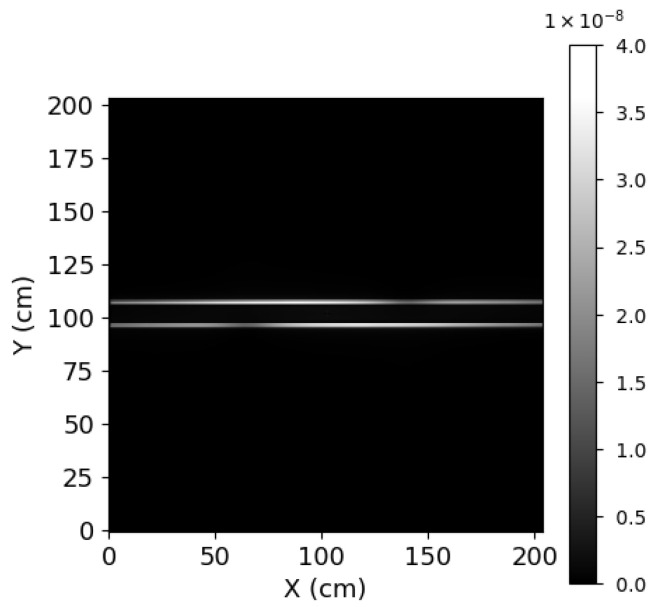
Two-dimensional power spectrum retrieved by the model.

**Figure 8:**
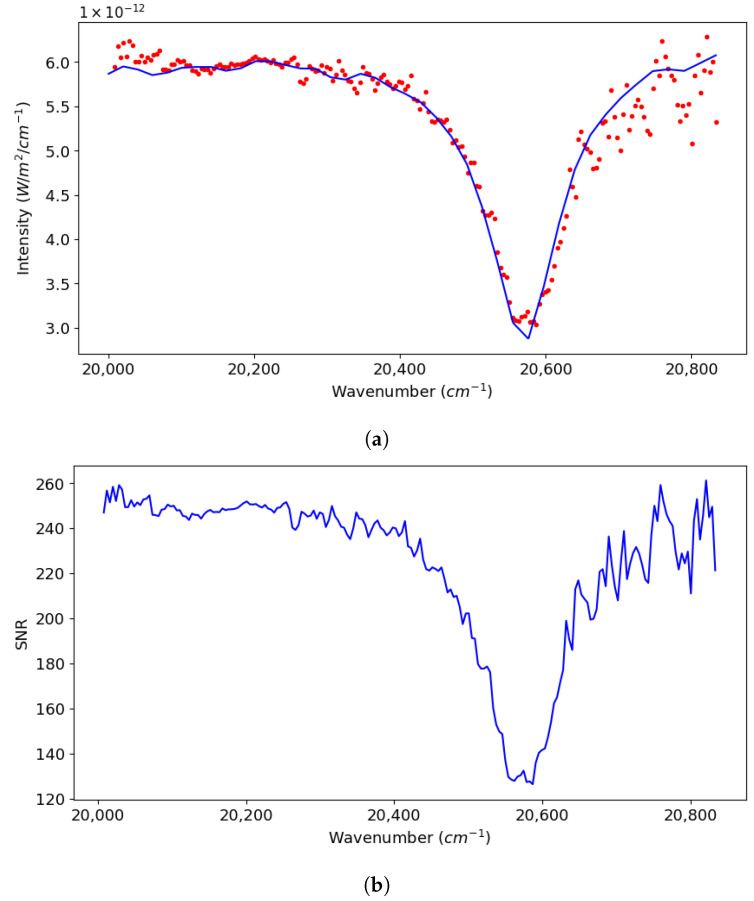
Spectra retrieval and data analysis. (**a**) Calibrated spectra along with input spectra, (**b**) signal-to-noise ratio.

Here, we show the spectral recovery achieved by our model SHS for a given use case, shown as a spectrum in (Figure 5). In (Figure 6), we have shown the processed and apodized 2D interferogram simulated by our SHS model for the input spectra and parameters listed above. In (Figure 7), we present the 2D power spectrum generated by carrying out an IFT on the simulated interferogram after removing any non-zero bias. We took the horizontal slice from the bottom half of this 2D spectrum and discarded the upper half, as it is just a mirror image of the bottom one. Finally, in (Figure 8), we present the results of our spectral recovery along with the original spectrum for validation. For the SNR calculation, we used the following formula, which was inspired by the work of Brault [28] and presented in the work of Harlander [29],

(26) $${\left(\frac{S}{N}\right)}_{k}=\frac{\sqrt{2}}{N}\frac{L_{k}}{L_{avg}}{\left(\frac{S}{N}\right)}_{x}$$

S/N denotes SNR, where the subscripts *x* and *k* correspond to the SNR in the spatial (or interferogram domain) and the spectral domains, respectively. *N* is the number of samples taken in the *x* or *y* directions on the detector used. $L_{k}$ and $L_{avg}$ correspond to the intensity of a spectral component *k* and the average spectral intensity in the spectra retrieved using the SHS model.

### 5.2. Model Optimization

**Figure 9:**
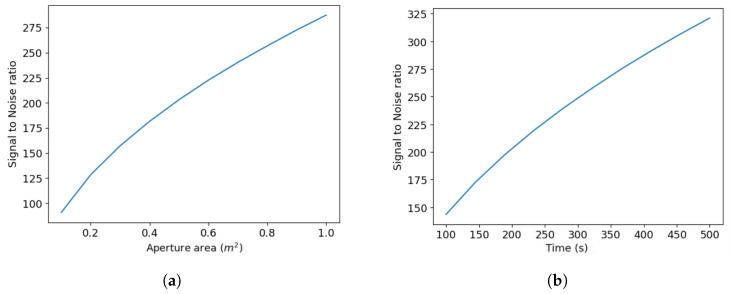
Design optimization for aperture area and exposure time, to understand the SNR dependence. (**a**) SNR as a function of the aperture area. As expected, this shows an increasing trend, due to increased photon counts, (**b**) SNR vs. exposure time also shows an increasing trend, due to the same reason as (**a**).

**Figure 10:**
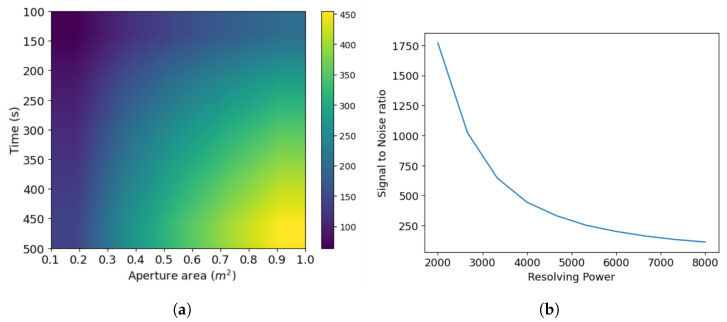
System optimization trade-offs. (**a**) SNR variation and heatmap to show dependence on area and time, (**b**) variation in SNR with resolving power. The decrease is due to the factor $\frac{1}{\sqrt{N}}$, where increasing N or the no. of samples decreases the resolving power. In addition, the photons are distributed amongst a greater number of pixels, thus increasing the error per pixel.

This study provides two crucial components to support the development of future SHS missions: an SHS model for simulating 2D interferograms, and an algorithm for processing interferograms. A useful model should be able to replicate certain real-life scenarios and give us a reference with which to work. We provide the results from our model optimization, to demonstrate that the model does this optimally. This involves choosing design parameters that effectively meet specific performance criteria. In this process, we focus on balancing aperture area (Figure 9a) and exposure time (Figure 9b) against SNR. We also plot a 2D heat map of SNR against area and time (Figure 10a) for the specific input parameters we discussed in the previous section, to facilitate the user in choosing the space and time according to their required SNR at a given resolving power.

We also look at other system trade-offs. The user must note that the SNR increases with decreasing R (other parameters are kept fixed) because a higher R relates to a higher number of samples, N, (Figure 10b), which means the same number of photons are distributed to a more significant number of samples, thus increasing the relative shot noise. Moreover, converting the SNR from the interferogram domain to the spatial domain involves an inverse relation in the number of samples N, as shown in Equation (26).

**Figure 11:**
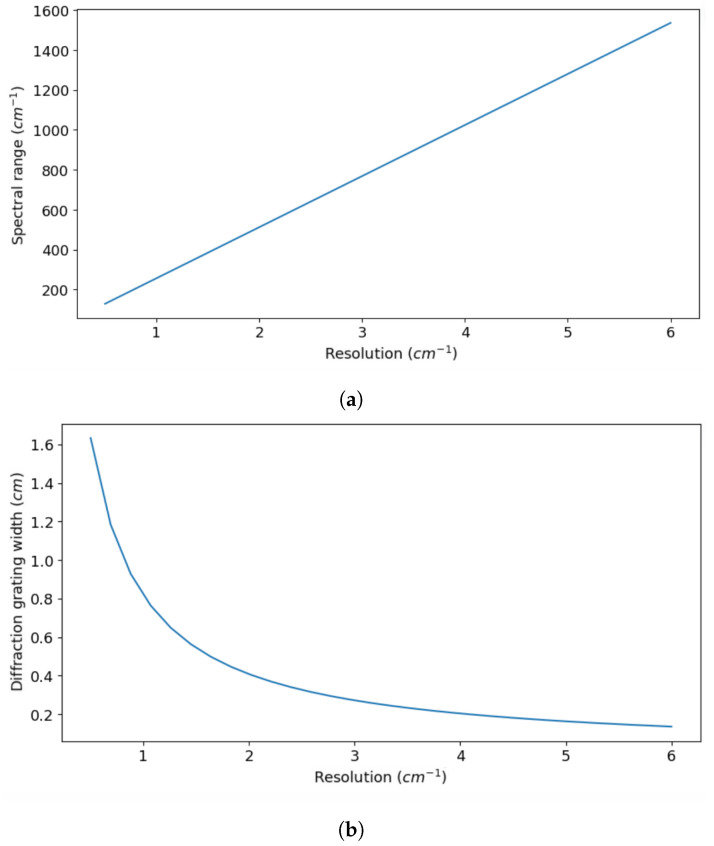
System optimization trade-offs. (**a**) Resolution as a function of the spectral range (Note: a lower resolution corresponds to better-resolving power). As we mentioned before, increasing the bandpass decreases the resolving capabilities of the SHS, (**b**) resolution as a function of the width of the diffraction grating (W). This shows a decreasing trend due to the relation R=4Wksinθ.

**Figure 12:**
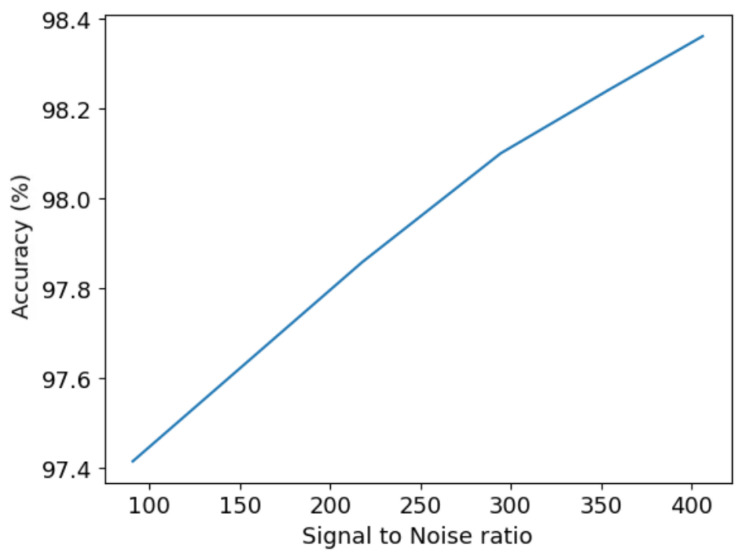
Results of the average spectrum recovery percentage accuracy as a function of SNR.

Next we look at trade-offs such as spectral range versus resolution (Figure 11a), diffraction grating width versus spectral resolution (Figure 11b). This aims to find the best parameters that achieve the desired performance, while considering different trade-offs in the system. The spectral resolution and range are inversely related (Figure 11a): a higher range can be more crucial in specific applications (like hyperspectral imaging for classification), while a higher resolution matters in studying emission or absorption lines. Increasing the resolution increases the number of samples and diffraction grating width (Figure 11b). This trade-off allows enhancing the resolution without sacrificing range but impacts instrument portability. Larger optical elements for a higher resolution conflict with the compactness and mechanical strength of the SHS design. Finally, (Figure 12) shows the average spectrum recovery percent accuracy concerning the SNR. This is valuable when aiming for a specific system accuracy rate, guiding the selection of the SNR for a certain integration time or aperture areas.This plot assists in determining the required SNR to achieve the desired system accuracy.

## 6. Science Cases for SHS

High-resolution spectroscopy utilizing SHS holds tremendous promise for studying specific objects and phenomena within the solar system, such as planetary atmospheres and satellites [2], as well as the interstellar medium (ISM) and exoplanet atmospheres. Many of these targets fall within the UV-visible range. SHS has the potential to emerge as a pivotal technology for their investigation, as outlined in the introduction. Furthermore, extending spectral analyses into the UV realm enhances information acquisition, as the solar continuum radiation drops rapidly in this region. This makes it very powerful for studying solar system targets and analyzing atmospheric emission lines, which would otherwise be difficult to analyze. The same goes for other stellar continuums and associated planetary system and their atmospheres [3]. Below, we briefly explore a range of SHS applications across different scientific contexts.

### 6.1. Atmospheric Science

**Figure 13:**
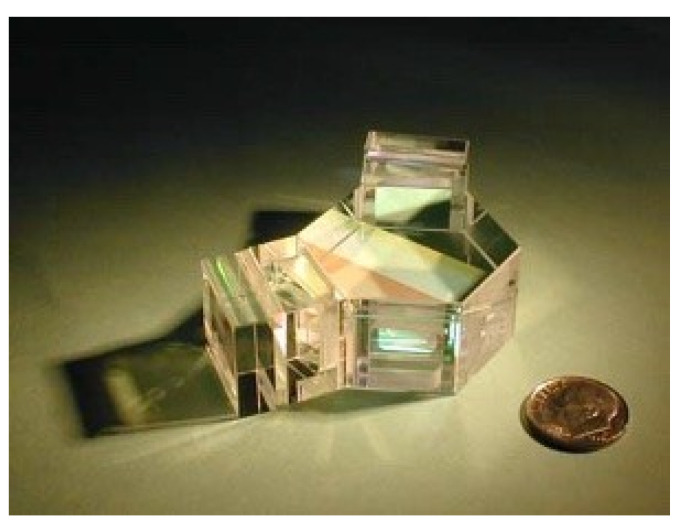
SHS interferometer for near-UV flown as part of the SHIMMER payload on STPSat-1 [39].

One of the key applications of SHS in atmospheric science lies in its ability to provide high-resolution spectra over a broad range of wavelengths. This feature allows for detailed atmospheric composition analysis, including identifying and quantifying trace gases, aerosols, and other crucial components. With its versatility and precision, SHS offers valuable insights into the complex interplay of atmospheric constituents. For example, after the conceptualization of an SHS instrument by Roesler and Harlander [15,18], the first instrument was built in the Naval Research Laboratory for an instrument known as SHIMMER (Spatial Heterodyne Imager for Mesospheric Radicals) [30,31] (Figure 13), which was the primary payload of the shuttle flight STS-112, 7–18 October 2002. It rivalled the contemporary Middle Atmosphere High-Resolution Spectrograph Investigation (MAHRSI) regarding the required resolution and smaller size for measuring mesospheric hydroxyl (OH) using solar resonance fluorescence in the near-infrared.

**Figure 14:**
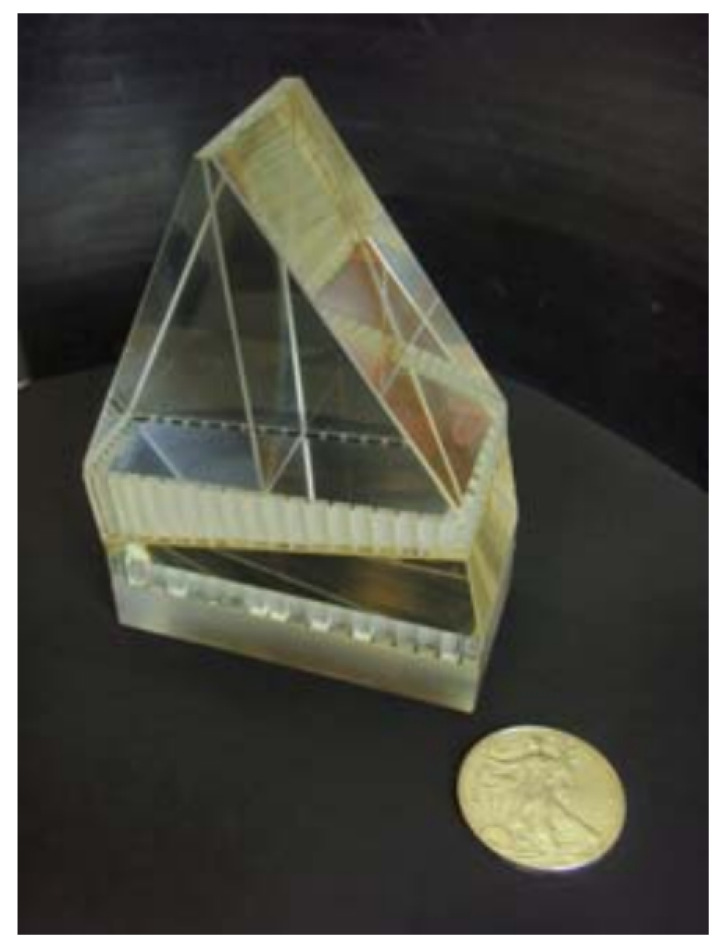
DASH interferometer designed for measuring thermospheric winds using the oxygen red line (λ = 630 nm) [17].

SHS technology can contribute to our understanding of air quality, climate change, and the impact of anthropogenic activities. SHS’s capacity to simultaneously capture multiple spectral lines enhances its utility for probing atmospheric dynamics. By studying the Doppler shifts and line shapes in observed spectra, SHS enables researchers to investigate wind patterns, temperature profiles, and other dynamic processes within the atmosphere. An important example is a modified SHS technique called DASH (Doppler asymmetric spatial heterodyne) spectroscopy, recently launched as a part of NASA’s ICON (Ionospheric Connection Explorer) mission. Initially conceptualized by Englert et al. [32,33], the instrument named MIGHTI (Michelson Interferometer for Global High-resolution Thermospheric Imaging) [34] (Figure 14) based on the DASH technology was launched in 2018. MIGHTI was designed to measure winds using the Doppler shifts of the atomic oxygen red (λ= 630 nm) and green (λ= 557.7 nm) emission lines and to measure temperature using the band shape of the molecular oxygen A-band around λ= 762 nm. DASH technology has also been used with LIDAR (light detection and ranging) [35] to meet the increasing need to acquire tropospheric wind fields, a priority for the NASA Earth Science Division. Another similar work was carried out by Kauffman et al. ([36]. It used limb sounding, which passively observes limb-scattered sunlight, using the Sun as a light source. SHOW (Spatial Heterodyne Observation of Water) already operates in the 1363–1366 nm range with a 0.03 nm spectral resolution, currently from an aeroplane [37]. Apart from these, Raman spectroscopy [38] can also be used in integration with SHS technology for high-resolution single molecule studies like H2O and CO2, which have applications in tracking pollutants in Earth’s atmosphere. Such information is instrumental in improving weather forecasting models and advancing our understanding of atmospheric circulation patterns.

Furthermore, SHS plays a significant role in studying the mesosphere and thermosphere, like the MIGHTI instrument, where traditional observational methods face challenges. The high spectral resolution of SHS allows for the precise measurement of temperatures and densities in these upper atmospheric regions, aiding in exploring phenomena such as atmospheric tides, gravity waves, and interactions with solar radiation.

### 6.2. Solar System Science

SHS is vital in investigating the interactions between solar wind and planetary magnetospheres. As discussed in the previous section, DASH technology holds immense importance in solar physics, principally in the subfield of space weather, magnetic field structures, and atmosphere dynamics, as well as thermospheric wind and temperature measurements. This is also highly relevant for NASA’s heliophysics roadmap [40], which has objectives like “What is responsible for the dramatic variability of the ionosphere thermosphere-mesosphere (ITM) region?” and “How do coupled middle and upper atmospheres respond to external drivers and each other?” [17]. Studying gravity waves in the mesosphere potentially holds the answers to these questions. SHS–DASH technology could be pivotal in understanding the spectral signatures associated with gravity waves. SHS can also satisfy the requirements of spatial imaging and spectral resolution for observing magnetic fields in solar flares. It would measure Zeeman splitting within a solar flare [41,42] using spectroscopy of infrared solar emission lines, which would give direct information on the local magnetic field within that flare and the conditions at its source, i.e., the Sun. Another niche for the SHS instrument may be UV spectro-polarimetric studies. This was discussed in detail by Harris et al., walterSHS. SHS has an advantageous all-reflective configuration that can significantly improve the UV optics compared to other instruments. Coupled with high etendue/throughput capabilities, SHS can significantly improve the noise problem other UV instruments face when studying diffused objects.

**Figure 15:**
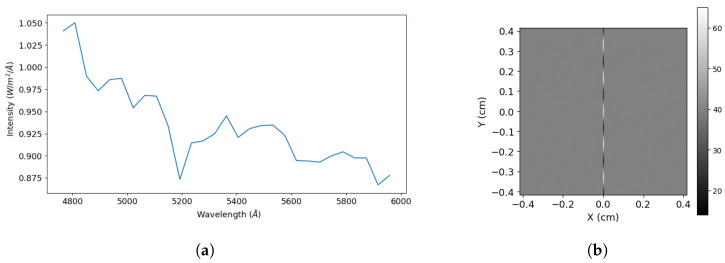
Model interferogram generated from a segment of solar spectra available at [43]. (**a**) We utilized the bandpass of 5000–5400 to generate a model interferogram, (**b**) interferogram generated by our SHS model.

**Figure 16:**
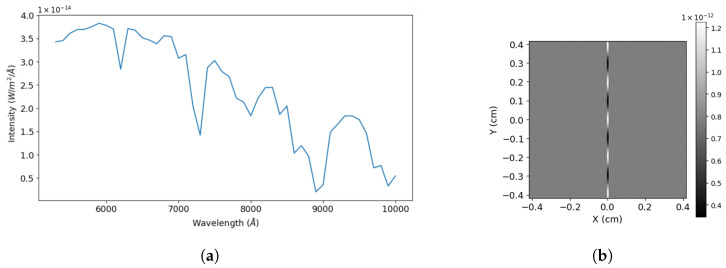
Model interferogram generated from a segment of Saturn surface brightness spectra available at [43]. (**a**) We utilized the bandpass of 6050–6350 to generate a model interferogram, (**b**) interferogram generated by our SHS model.

One of the other primary focuses of SHS in Solar System exploration lies in the detailed study of planetary atmospheres. For example, similarly to Earth’s atmosphere, limb sounding of the atmosphere of other planets like Mars can also be performed to identify CH4, H2O, and CO2 and their precise volume mixing ratios. By examining spectral features in the ultraviolet and infrared regions, SHS provides a comprehensive understanding of the composition, temperature profiles, and dynamic processes within the atmospheres of planets, moons, and other celestial bodies. We present some model interferograms for solar system objects in Figure 15 and Figure 16. Figure 15a simulates a 2D interferogram for the segment of the solar spectra shown in Figure 15b and provides corresponding input and output parameters in Table 4 and Table 5.

Similarly, in Figure 16 and Table 6 and Table 7, the same has been carried out for a segment of the spectra of Saturn (Figure 16a).

In the realm of planetary rings, SHS technology can stand out as a powerful tool for investigating the composition and structure of ring systems. By analyzing the spectral signatures of ring particles, SHS will facilitate a deeper understanding of the dynamics, particle sizes, and material properties within planetary ring systems. This knowledge is crucial for deciphering the formation and evolution of ring structures around gas giants like Saturn and ice giants such as Uranus and Neptune.

#### Cometary Spectrophotometry

Continuing the discussion, SHS can also excel in studying cometary and asteroidal environments. The composition of comets contains information about the early solar system’s environment, dust, and temperatures. By capturing spectra from and around these celestial objects, SHS enables the identification of essential chemical compounds, shedding light on the origin and evolution of these primitive bodies and the solar system as a whole. Many essential chemical bands like OH and CO lie in the UV regime, where SHS has a significant advantage over other instruments. Variation in composition among comets (broadly divided into two classes: Jupiter family and Oort family) [44] is mainly the result of differences in formation conditions and weathering. The neutral coma of comets presents us with a unique opportunity to study the properties of gasses that are difficult to study in the laboratory [19]. Owing to low gas densities, the chemical reactions among species are rare, providing the scope to explore species like OH.

LIBS (laser-induced breakdown spectroscopy) [45], an active method like Raman spectroscopy, where lasers are used rather than other monochromatic sources, can be integrated with SHS technology [46]. This uses a high-energy laser beam pointed at the study sample, causing the vaporization and atomization of the components. The atoms are then excited and ionized in a high-temperature plasma. The excited atoms and ions return to their initial state by emitting photons, the energy of which corresponds to the natural emission spectrum of the atom. Such a method can allow measuring the quantities of different elements in a solid sample, such as comets and asteroids.

### 6.3. Interstellar Medium

SHS’s high spectral resolution capabilities and large throughput compared to conventional satellites make it a powerful tool to significantly advance our understanding of the ISM. A primary application of SHS lies in its ability to map the distribution and dynamics of molecular clouds within the ISM. By capturing spectral lines associated with molecules such as carbon monoxide (CO) and ammonia (NH3), SHS unveils the structural complexities of molecular clouds, providing invaluable insights into the fundamental processes of star formation and the gravitational interactions shaping these cosmic regions.

For example, SHS has already been used to analyze the O II line at λ = 372.7 nm in the warm ionized medium (10,000 K) of the ISM, supposedly caused by heating from unknown sources [47,48,49,50]. Another mission has been outlined by Hosseini et al. [51], which aims at measuring the spectral lines corresponding to the C IV doublet at λ = 155 nm. This would help us understand the fundamentals of stellar life cycles. Another recurring mystery in the context of ISM has been the hot region, which has temperatures of $10^{5}$–$10^{7}$ K. Its influence on the structure and dynamics of the interstellar medium and galactic halo has long been debated. The reason for this is the unavailability of spectroscopic instruments with sufficient resolution and sensitivity to simultaneously analyze the hot component’s distribution and kinematics [49]. SHS technology was employed to solve this problem due to its resolution and throughput capabilities, but the results were mostly non-conclusive, due to contamination of the optical elements [50].

In addition to its role in characterizing molecular and ionized components, SHS provides a unique perspective on the turbulent nature of the ISM. By observing spectral signatures associated with shockwaves and turbulent motions, SHS contributes to understanding the energy dissipation processes, magnetic field interactions, and overall dynamics shaping the interstellar medium. The integration of SHS into space-based observatories, coupled with collaborative efforts with other instruments across different wavelengths, is poised to push the frontiers of ISM research.

## 7. Discussions

High-resolution spectroscopy using SHS has excellent potential for future space missions, leveraging its past success. Principally, it promises to perform exceptionally well and economically cheaper than other contemporary instruments in the UV-Vis region. This study introduced the SHS model, the generation of 2D interferograms, and a processing algorithm to aid SHS research. It offered analysis and design optimization tools, demonstrating accurate spectral recovery with realistic SNR values. Our model also web-scrapes from the Pickles Atlas [26], so that the user can choose stellar sources of the desired spectral type and derive required SHS parameters for realistic results. To demonstrate its usability, we compared the results from our SHS model with the previously flown SHS instrument SHIMMER [30]. The input parameters for the SHIMMER instrument are shown in Table 8 and in Table 9 we present the actual and model-derived instrument parameters. The results show that the simulated SHIMMER instrument parameters match closely with the original instrument, as described in the literature [30].

There is additional potential for improvement in the proposed model. Presently, our model generates the SNR based on specified parameters. However, its utility could be significantly enhanced by enabling it to generate parameters tailored to achieve the desired SNR for specific targets. Additionally, a future iteration of this model could incorporate consideration of phase errors arising from slight misalignments in the interferogram, thereby yielding a more realistic SHS model. Furthermore, there exists an opportunity to explore alternative SHS configurations such as field-widened SHS and all-reflective SHS, which hold promise, particularly in ultraviolet (UV) research.

We have also presented a wide variety of ongoing and potential future missions that the SHS instrument is involved in, to cater to the continuing development of the field. Future scientific missions, be it for environmental monitoring or astrophysical studies, will benefit from these instruments, which could even be deployed on CubeSats or as secondary payloads on host spacecraft.

Furthermore, SHS plays a significant role in studying the mesosphere and thermosphere, where traditional observational methods face challenges. The high spectral resolution of SHS allows for the precise measurement of temperatures and densities in these upper atmospheric regions, aiding in exploring phenomena such as atmospheric tides, gravity waves, and interactions with solar radiation. SHS extends its applicability to space missions exploring other celestial bodies in the context of planetary atmospheres beyond Earth. Its ability to analyze the atmospheres of planets, moons, and comets contributes to our broader understanding of planetary evolution, climate variations, and the potential habitability of distant worlds. As SHS technology continues to evolve, its integration into satellite-based platforms and ground-based observatories as a compact and lightweight tool, enabled with high etendue and resolving power, promises to enhance our observational capabilities in atmospheric science.The deployment of SHS in combination with other instruments offers a synergistic approach, enabling more comprehensive studies of Earth’s atmosphere and expanding our exploration of planetary atmospheres within our solar system and beyond.

## Figures and Tables

**Table 1:** Here, we present a comparison of various spectroscopic instruments with SHS at its theoretical maximum collimation [19] on a supposed cubesat. Abbreviations: FUSE—Far Ultraviolet Spectroscopic Explorer, STIS—Space Telescope Imaging Spectrograph, UVIS—Uv imaging spectrograph.

Instrument	Aperture (${\mathrm{cm}}^{2}$)	Ω (sq. deg.)	Etendue	Resolving Power
SHS	50	0.2	10	$10^{5}$
FUSE (LiF ch1)	1360	7 × $10^{-5}$	0.09	$10^{4}$
HST-STIS (E140H)	45,000	3 × $10^{-7}$	0.0135	$10^{4}$–$10^{5}$
Cassini UVIS	6	1.24	7.5	<<1000

**Table 2:** Input parameters provided as a use case.

Input Parameters	Values
Range (Angstrom)	4800–5000
resolving Power	5000
d (grating wedge spacing) (cm)	0.001
m (order)	3
A (aperture area) ($m^{2}$)	0.3
integration Time (s)	400

**Table 3:** Output parameters generated by the model using the input parameters.

Output Parameters	Values
Liitrow wavenumber ($k_{L}$) (${\mathrm{cm}}^{-1}$)	20,417
N × N (number of interferogram samples required)	204 × 204
theta (Littrow angle) (°)	4.21
W (grating width) (cm)	0.83
dk (resolution achieved at central wavelength) (${\mathrm{cm}}^{-1}$)	4.08

**Table 4:** Input parameters provided as a use case for the Solar spectra shown in Figure 15a.

Input Parameters	Values
Range (Å)	5000–5400
Resolving Power	5000
d (grating wedge spacing) (cm)	0.001
m (order)	3
A (aperture area) ($m^{2}$)	0.3
Integration Time (s)	400

**Table 5:** Output parameters generated by the model using input parameters for the solar spectra.

Output Parameters	Values
Littrow wavenumber ($k_{L}$) (${\mathrm{cm}}^{-1}$)	19,230.77
N × N (number of interferogram samples required)	328 × 328
Theta (Littrow angle) (°)	4.46
W (grating width) (cm)	0.83
dk (resolution achieved at central wavelength) (${\mathrm{cm}}^{-1}$)	3.86

**Table 6:** Input parameters provided as a use case for the Saturn spectra shown in Figure 16a.

Input Parameters	Values
Range (Å)	6050–6350
Resolving Power	5000
d (grating wedge spacing) (cm)	0.001
m (order)	3
A (aperture area) ($m^{2}$)	0.3
Integration Time (s)	400

**Table 7:** Output parameters generated by the model using input parameters for the Saturn spectra.

Output Parameters	Values
Littrow wavenumber ($k_{L}$) (${\mathrm{cm}}^{-1}$)	16,129.03
N × N (number of interferogram samples required)	162 × 162
Theta (Littrow angle) (°)	5.34
W (grating width) (cm)	0.84
dk (resolution achieved at central wavelength) (${\mathrm{cm}}^{-1}$)	3.21

**Table 8:** Input parameters derived from SHIMMER that are used in our 2D SHS model.

Input Parameters	Values
Range (Å)	3055.5–3084.5
Resolving power	53,500
d (grating wedge spacing) (cm)	0.00083
m (order)	1

**Table 9:** Comparison of output parameters of our 2D SHS model with respect to SHIMMER parameters. The N × N parameter for SHIMMER has been left blank as it was not mentioned in the original paper by Harlander et al. [30].

Output Parameters	SHIMMER	2D SHS Model
Littrow wavelength ($\Lambda_{L}$) (Å)	3070	3070
N × N (number of interferogram samples required)	—	506 × 506
theta (littrow angle) (°)	10.7	10.66
W (grating width) (cm)	2	2.22
dΛ (resolution achieved) (Å)	0.058	0.057

## Data Availability

The data can be provided upon request.
